# Amino Acid and Secondary Metabolite Production in Embryogenic and Non-Embryogenic Callus of Fingerroot Ginger (*Boesenbergia rotunda*)

**DOI:** 10.1371/journal.pone.0156714

**Published:** 2016-06-03

**Authors:** Theresa Lee Mei Ng, Rezaul Karim, Yew Seong Tan, Huey Fang Teh, Asma Dazni Danial, Li Sim Ho, Norzulaani Khalid, David Ross Appleton, Jennifer Ann Harikrishna

**Affiliations:** 1 Centre for Research in Biotechnology for Agriculture & Institute of Biological Sciences, Faculty Science, University of Malaya, 50603 Kuala Lumpur, Malaysia; 2 Sime Darby Technology Centre, 1st Floor Block B, UPM-MTDC Technology Centre III, Universiti Putra Malaysia, 43400, Serdang, Selangor, Malaysia; 3 Department of Botany, Faculty of Life and Earth Sciences, University of Rajshahi, Rajshahi 6205, Bangladesh; Indian Institute of Chemical Technology, INDIA

## Abstract

Interest in the medicinal properties of secondary metabolites of *Boesenbergia rotunda* (fingerroot ginger) has led to investigations into tissue culture of this plant. In this study, we profiled its primary and secondary metabolites, as well as hormones of embryogenic and non-embryogenic (dry and watery) callus and shoot base, Ultra Performance Liquid Chromatography-Mass Spectrometry together with histological characterization. Metabolite profiling showed relatively higher levels of glutamine, arginine and lysine in embryogenic callus than in dry and watery calli, while shoot base tissue showed an intermediate level of primary metabolites. For the five secondary metabolites analyzed (ie. panduratin, pinocembrin, pinostrobin, cardamonin and alpinetin), shoot base had the highest concentrations, followed by watery, dry and embryogenic calli. Furthermore, intracellular auxin levels were found to decrease from dry to watery calli, followed by shoot base and finally embryogenic calli. Our morphological observations showed the presence of fibrils on the cell surface of embryogenic callus while diphenylboric acid 2-aminoethylester staining indicated the presence of flavonoids in both dry and embryogenic calli. Periodic acid-Schiff staining showed that shoot base and dry and embryogenic calli contained starch reserves while none were found in watery callus. This study identified several primary metabolites that could be used as markers of embryogenic cells in *B*. *rotunda*, while secondary metabolite analysis indicated that biosynthesis pathways of these important metabolites may not be active in callus and embryogenic tissue.

## 1. Introduction

*Boesenbergia rotunda* is a member of the Zingiberaceae family. This monocotyledon plant is commonly called fingerroot, Chinese keys or Chinese ginger and is used in food, flavourings and traditional medicines [1]. Several flavonoids and chalcone derivatives have been isolated from extracts of *B*. *rotunda*, including pinocembrin, pinostrobin, alpinetin, panduratin, cardamonin, quercetin and kaempferol [2]. These compounds are reported to have various biological effects. Kirana *et al*. reported that a concentration of 9.0 μg.mL^-1^ panduratin A completely inhibited the growth of MCF-7 human breast cancer cells and HT-29 human colon adenocarcinoma cells [3]. Pinostrobin extracted from the rhizomes of *B*. *rotunda* has been reported to show anti-microbial [4], anti-ulcer [5], anti-viral [6] and anti-tumor [7] activity. Rhizomes and other parts of the plant have also been used to investigate the various biological activities. Jing *et al*. compared its anti-proliferative effect against five cancer cell lines using extracts from rhizomes, leaves and stems from different *Boesenbergia* species [8]. They found that extracts from the rhizome of *B*. *rotunda* gave the most promising results in cytotoxic activity for all five cancer cell lines.

*B*. *rotunda*’s various beneficial effects have spurred extensive study of tissue culture of this plant. Mass propagation via tissue culture not only saves time but also yields clonal plant materials that can be manipulated through culture conditions or genetic engineering to produce desirable metabolites. It has long been known that biomass yields and metabolite production are influenced by physiology and plant development. Specifically in *B*. *rotunda*, biomass yield has been enhanced via manipulation of physical cell culture conditions [9] and usage of various concentrations of plant growth regulators such as 2,4-dichlorophenoxyacetic acid (2,4-D) and 6-benzyl amino purine (6-BAP), which have been applied to increase the rates of cell proliferation and somatic embryogenesis [10–12].

Manipulation of cell culture conditions to increase embryogenesis rates can be expected to affect metabolism and thus might also have an impact on the production of specific metabolites of interest. One way to investigate cell metabolism in relation to embryogenesis is through omics technologies such as genomics, transcriptomics, proteomics and metabolomics in order to observe the underlying molecular changes during tissue culture [13–16]. We characterized the shoot base, embryogenic and non-embryogenic calli (dry and watery) of *B*. *rotunda* using metabolite profiling to probe the underlying biochemical processes associated with embryogenesis. Targeted metabolites in various tissue types, including primary and secondary metabolites as well as hormones were analyzed using Ultra Performance Liquid Chromatography–Mass Spectrometry (UPLC-MS). Furthermore, to relate biochemistry to morphology, microscopy analyses were performed on the callus and shoot base.

## 2. Materials and Methods

### 2.1 Ethics statement

The conduct of this research was approved by the grant management committee of the University of Malaya, headed by the Director of Institute of Research Management and Monitoring, Professor Noorsaadah Abdul Rahman (noorsaadah@um.edu.my). This study did not involve the use of any human, animal and endangered or protected plant species as materials and the study did not include any field study or site study.

### 2.2 Plant source

*B*. *rotunda* rhizomes were purchased from a commercial herb farm in Pahang, Malaysia and propagated in the laboratory to generate all sample materials. Initially, the plants were washed thoroughly under running tap water for 10 min, then air dried for 30 min before insertion into black polybags to promote sprouting. Samples were sprayed with water every day to induce growth of shoots. Newly formed shoots of less than 5 cm length were harvested for subsequent culture and analysis. Concurrently, additional shoots were allowed to grow to a length of 10 cm and were harvested as 5 cm long shoot samples which we labeled as T1: 1–5 cm portion of the shoot and T2: 6–10 cm portion of the shoot.

### 2.3 Establishment of tissue culture callus

Callus materials were established in three steps: sterilization, explant preparation and callus induction. First, shoots were collected and cleaned thoroughly with tap water. Next, the leaves of the outer layer were removed and the exposed tissues were sterilized with 20% Clorox and Tween-20 for 10 min. Next, the tissues were washed with 95% ethanol followed by thrice rinsing with deionized water. The sterilized tissue was dried on a clean filter paper. Then, a 1 mm cross-section from the shoot base (SB) tissue, including the shoot meristem, was cut and placed into callus induction media comprising a Murashige and Skoog base supplemented with 1 mg.L^-1^ α-napthaleneacetic acid (NAA), 1 mg.L^-1^ indole-3-acetic acid (IAA), 30 g.L^-1^ sucrose and 2 g.L^-1^ Gelrite^®^ (Sigma Aldrich, Missouri, United States). The callus that formed was transferred to a propagation medium containing 30 g.L^-1^ sucrose, 2 g.L^-1^ Gelrite^®^ and various concentrations of 2,4-dichlorophenoxy acetic acid (2,4-D) as follows; for dry callus (DC) (4 mg.L^-1^), for embryogenic callus (EC) (3 mg.L^-1^) and for watery callus (WC) (1 mg.L^-1^) [10, 12]. Enrichment of embryogenic cells from embryogenic callus was performed by sieving embryogenic calli through a 425 μm stainless steel sieve prior to extraction of metabolites.

### 2.4 Metabolite extraction protocols

#### Primary and secondary metabolites

Rhizome, shoot tissue and calli samples each had three biological replicates. The samples were ground to a fine power under a stream of liquid nitrogen. Fine powdered samples weighing 200 mg each were used for the extraction process. For shoot tissue, the shoot base and two samples (T1: 1–5 cm and T2: 6–10 cm as described above) were included for secondary metabolite analysis. The samples were extracted according to the method reported by Neoh *et al*. [17]. Concentrations were normalized using an internal standard according to dry weight of extract for each tissue type obtained.

#### Hormones

Hormone classes analyzed included cytokinins, gibberellins, auxins, salicylates, jasmonates and abscisic acid (ABA). Shoot base tissue and calli each had three biological replicates. Fine powdered samples weighing 100 mg were extracted using 1 mL of methanol (MeOH)/isopropanol (20/80, v/v) mixture with 1% (v/v) glacial acetic acid. Next 1950 *g* of the mixture was sonicated at 37 kHz in an Elmasonic S120H (Singen, Germany) for 20 min at 4°C to 7°C. Then, the mixture was placed into a centrifuge for 5 min at 4°C. The supernatant was transferred into a new clean tube. The process of extraction was repeated twice, each with fresh solvent mixture added [18]. All the supernatants were combined and dried using a Genevac (Ipswich, United Kingdom) evaporator.

### 2.5 Chemicals and reagents

Both primary and secondary metabolite standards were purchased from Sigma Aldrich (Missouri, United States) except for panduratin standard, which was obtained from in-house isolation (Prof Rais Mustafa, Faculty of Medicine and Dr. Lee Y.K, Faculty of Science, University of Malaya). The hormone standards were purchased from OlChemIm Ltd. (Olomouc, Czech Republic). These standards were used as is.

### 2.6 Analysis using Ultra Performance Liquid Chromatography-Mass Spectrometry (UPLC-MS)

#### Primary metabolites

Dry extracts were first dissolved in 100 μL 50% acetonitrile (ACN). Dry extracts were analyzed in triplicate using a Waters Acquity (Massachusetts, United States) LC system coupled with a Xevo Triple Quadrupole Mass Spectra (Massachusetts, United States) detector. The separation was performed using an Acquity UPLC^®^ HSS T3 column (1.8 μm, 2.1 mm x 100 mm) with solvent A [0.1% formic acid (FA) in water (H_2_O)] and solvent B (0.1% FA in ACN), according to the protocol. The elution gradient was as follows: initial at 95% solvent A; 0–3 min linear gradient to 60% solvent A; 3–5 min linear gradient to 5% solvent A; 5.0–5.1 min linear gradient to 95% solvent A and hold to 7 min. The flow rate was set to 0.3 mL.min^-1^ with an injection volume of 3 μL. Both positive and negative electron spray ionization (ESI) modes were used in the mass detector with a desolvation temperature of 350°C and capillary voltage at 2.9 kV. The total acquisition time was 15 min. The mass spectrometry parameters were optimized for detection of each metabolite using multiple reaction monitoring (S1 Table). Data were quantified in relative abundance against an internal standard.

#### Secondary metabolites

Five secondary metabolites of interest, namely panduratin, pinocembrin, pinostrobin, alpinetin and cardamonin were chosen because of reported biological activities as well as readily available standards. Dry extracts were first dissolved in 100 μL 50% acetonitrile (ACN). Dry extracts were analyzed in triplicate using a Waters Acquity (Massachusetts, United States) LC system coupled with a Xevo Triple Quadrupole Mass Spectra (Massachusetts, United States) detector. The separation was performed using Acquity UPLC^®^ BEH C18 column (1.7 μm, 2.1 mm x 100 mm) with corresponding solvent A (0.1% FA in H_2_O) and solvent B (0.1% FA in ACN). The elution gradient was as follows: initial at 60% solvent A; at 0–10 min linear gradient to 10% solvent A and hold to 2 min; 12–12.5 min linear gradient to 60% solvent A and hold to 2.5 min. The flow rate was set to 0.3 mL.min^-1^ with an injection volume of 3 μL. Positive ESI mode was used in the mass detector with desolvation temperature of 350°C while the capillary voltage was set to 3.5 kV. The total acquisition time was 15 min. The mass spectrometry parameters were optimized for detection of each metabolite using multiple reaction monitoring (S1 Table). Calibration curves for each standard were prepared and data were quantified in percent dry extracts and wet weight.

#### Hormones

Dry extracts were first dissolved in 100 μL 50% methanol (MeOH) and analyzed in triplicate using a Waters Acquity (Massachusetts, United States) LC system coupled with a Xevo Triple Quadrupole Mass Spectra (Massachusetts, United States) detector. The separation was done using a UPLC^®^ HSS T3 column (1.8μm, 2.1 mm x 100 mm) with corresponding solvent A (0.1% FA in H_2_O) and solvent B (0.1% FA in MeOH). The elution gradient was as follows: initial at 99.9% solvent A; at 0–3 min linear gradient to 70% solvent A; 3–8 min linear gradient to 100% solvent B and hold to 2 min; 10–13 linear gradient to 70% solvent A; 13–14 min linear gradient to 99.9% solvent A and hold to 1 min. The flow rate was set to 0.25 mL.min^-1^ with an injection volume of 3 μL. Both positive and negative ESI mode were used in the mass detector with desolvation temperature of 330°C while the capillary voltage was set to 4.5 kV. The total acquisition time was 10 min. The mass spectrometry parameters were optimized for detection of each metabolite using multiple reaction monitoring (S1 Table). Calibration curves for each standard were prepared and data were quantified in parts per million.

### 2.7 Statistical analysis

Data from MS were processed using Target Lynx^™^ software (Waters, Massachusetts United States). In addition, clustering analysis was performed using Principal Component Analysis (PCA) and Orthogonal Partial Least Square Analysis (OLPS-DA) by Umetrics (Malmo, Sweeden) using Simca-P (Version 13). Moreover, ANOVA by IBM SPSS Statistics (Version 20) and t-test algorithm of Excel 2000 by Microsoft analysis were performed to identify significant differences with a 95% confidence level.

### 2.8 Histology

#### Scanning electron microscopy

Samples were fixed using 4% glutaraldehyde for 2 days at 4°C followed by washing with 0.1 M sodium cacodylate buffer at intervals of 30 min (repeated 3 times). Next, the samples were post-fixated with 1% osmium tetroxide for 2 h at 4°C. Subsequently, samples were washed again three times with 0.1 M sodium cacodylate buffer for 30 min each before the dehydration process using a series of acetone water mixtures (35% acetone, 50%, 75% and 95% acetone) for 45 min each. After that, the samples were incubated in 100% water for an hour (repeated three times). Samples were then dried in a Bal-Tec CPD 030 (Schalksmuhle, Germany) critical point dryer at 40°C for 90 min, mounted on stubs and gold coated before viewing. Finally, samples were examined under a Jeol JSM-6400 scanning electron microscope, with X-ray analyzer.

#### Semi-thin sections

Samples were fixed using glutaraldehyde-paraformaldehye-caffeine solution containing 50% (v/v) 0.2 M phosphate buffer (pH 7.2), 4% (v/v) glutaraldehyde, 20% (v/v) paraformaldehyde and 10 g.L^-1^ caffeine in distilled water. The samples were then dehydrated using different ethanol-water percentages; [30% ethanol for 30 min; 50%, 70% ethanol (each for 45 min); 80% ethanol, 90% and 95% (each for an hour)]. After that, the samples were incubated in 100% water for an hour (repeated twice). Next, the samples were infiltrated and embedded with Technovit^®^ 7100 (Hanau, Germany) resin prior to mounting. Semi-thin sections (3.5 μm) were prepared by microtome.

#### Light microscopy

Semi-thin sections were stained with Periodic acid-Schiff reagent before examination under light microscopy using an Olympus BX51 model. Estimation of the number of cells per unit area in each sample was performed using the analySIS FIVE LS Research (Version 5) software by Olympus Soft Imaging Solutions, (Munster, Germany).

#### Fluorescence microscopy

Semi-thin sections were stained with diphenylboric acid 2-aminoethylester (DPBA) for 15 min before viewing under an Olympus BX51 model fluorescent microscope with excitation and emission wavelengths of 400–410 nm and 455 nm, respectively (U-MNV2 mirror unit).

## 3. Results and Discussion

### 3.1 Primary metabolite analysis

We have determined the relative abundance of fifty-one targeted primary metabolites in shoot base and three callus types (embryogenic callus, dry callus and watery callus) of *B*. *rotunda* using UPLC-MS (Table 1). Data were normalized based on dry extract weight of tissues, although a similar trend could be observed when normalization was performed using the estimated cell density (cells/mm^2^) for each sample (Table 2). Embryogenic callus (EC) had the highest abundance of most primary metabolites, followed by shoot base (SB). Comparatively, both watery (WC) and dry (DC) callus had significantly lower levels of primary metabolites. In particular, EC was observed to have comparatively high levels of amino acids. This may be due to higher amino acid requirement for cell differentiation and division leading to plant regeneration. For example, phenylalanine and tryptophan are precursors for secondary metabolite and hormone metabolism and were observed to be approximately 15 times more concentrated in EC compared to SB. Furthermore, proline has also been reported as one of the amino acids used to induce maturation of somatic embryogenesis in strawberry [19]. In contrast, the high organic acid levels in SB tissue may be closely related to its role in energy production in the form of adenosine triphosphate (ATP). The shoot base samples contained meristem cells that are capable of differentiation into organs and self-multiplication. Thus, SB cells are actively involved in metabolism of carbohydrates, fats and proteins to form the ATP needed for growth. A transcriptomics study in maize showed up-regulated transcripts in ATP synthesis in the newly formed shoot meristem, compared to mature meristem tissue [20].

**Table 1:** Relative abundance of primary metabolites and their associated pathways in shoot base, embryogenic and non-embryogenic calli in *B*. *rotunda*.

Metabolites	Pathways	SB	EC	DC	WC
**Glycine (Gly)**	**Amino acid**	ND	0.0037 ± 0.0017	ND	0.00073 ± 0.00018
**Homoserine**	**Amino acid**	0.80 ± 0.27	1.62 ± 0.29	0.00274 ± 0.00079	0.026 ± 0.020
**Glutamine (Gln)**	**Amino acid**	27.7 ± 3.9	130 ± 26	0.0551 ± 0.0036	0.161 ± 0.065
**Histidine (His)**	**Amino acid**	5.2 ± 1.5	94 ± 19	0.0238 ± 0.0094	0.139 ± 0.065
**S-adenosyl methionine**	**Amino acid**	0.29 ± 0.13	2.84 ± 0.37	ND	ND
**Spermine**	**Amino acid**	ND	0.42 ± 0.20	0.0070 ± 0.0018	0.0138 ± 0.0024
**Arginine (Arg)**	**Amino acid**	10.0 ± 3.3	370 ± 69	0.070 ± 0.017	0.55 ± 0.31
**Alanine (Ala)**	**Amino acid**	ND	0.467 ± 0.088	ND	0.0020 ± 0.0003
**Asparagine (Asn)**	**Amino acid**	0.81 ± 0.38	0.43 ± 0.15	ND	0.0184 ± 0.0016
**Aspartic acid (Asp)**	**Amino acid**	5.9 ± 2.7	3.75 ± 0.93	0.00513 ± 0.00087	0.065 ± 0.047
**Glutamic acid (Glu)**	**Amino acid**	23.4 ± 6.7	2.44 ± 0.27	0.028 ± 0.077	0.25 ± 0.14
**Serine**	**Amino acid**	ND	0.0169 ± 0.0051	ND	ND
**Proline (Pro)**	**Amino acid**	0.28 ± 0.14	3.06 ± 0.66	0.0013 ± 0.0002	0.015 ± 0.012
**Phenylalanine (Phe)**	**Amino acid**	0.116 ± 0.055	1.48 ± 0.35	ND	0.01544 ± 0.00065
**Valine (Val)**	**Amino acid**	0.96 ± 0.21	7.6 ± 1.6	0.0187 ± 0.0029	0.049 ± 0.045
**Tyrosine (Tyr)**	**Amino acid**	0.7 ± 0.29	4.22 ± 0.82	0.0091 ± 0.0024	0.0075 ± 0.0010
**Trptophan (Trp)**	**Amino acid**	1.7 ± 0.4	26.7 ± 7.0	ND	ND
**Hydroxyproline**	**Amino acid**	ND	0.170 ± 0.036	ND	0.002601 ± 0.000083
**Lysine (Lys)**	**Amino acid**	47.5 ± 7.1	190 ± 33	0.0676 ± 0.0068	0.24 ± 0.12
**Methionine (Met)**	**Amino acid**	ND	0.036 ± 0.013	ND	ND
**Antranilate**	**Amino acid**	0.083 ± 0.026	5.08 ± 0.83	ND	0.0116 ± 0.0023
**Adenine**	**Amino acid**	0.24 ± 0.13	2.06 ± 0.47	0.00461 ± 0.00081	0.0066 ± 0.0027
**Creatine**	**Amino acid**	ND	0.0086 ± 0.0028	ND	0.0050 ± 0.0027
**Glycerol-3-phosphate**	**Glycolysis**	1.5 ± 1.4	3.2 ± 1.1	0.048 ± 0.035	0.118 ± 0.045
**Fructose-6-phosphate**	**Glycolysis**	12.1 ± 4.3	22 ± 14	0.40 ± 0.19	0.70 ± 0.34
**Fructose-1,6-phosphate**	**Glycolysis**	0.180 ± 0.037	0.46 ± 0.15	0.073 ± 0.071	0.059 ± 0.037
**Gluconic acid**	**Pentose Phosphate**	0.53 ± 0.32	0.79 ± 0.36	0.19 ± 0.11	0.27 ± 0.17
**Erythrose-4-phosphate**	**Pentose Phosphate**	0.21 ± 0.09	0.38 ± 0.25	0.0196 ± 0.0054	0.0383 ± 0.020
**Xylulose-5-phosphate**	**Pentose Phosphate**	0.205 ± 0.089	0.32 ± 0.17	ND	0.0096 ± 0.0074
**Ribulose-5-phosphate**	**Pentose Phosphate**	0.73 ± 0.41	1.20 ± 0.46	ND	0.0208 ± 0.0095
**6-phosphogluconic acid**	**Pentose Phosphate**	0.500 ± 0.068	ND	0.46 ± 0.31	0.97 ± 0.70
**Putresine**	**Polyamines**	ND	0.043 ± 0.011	ND	ND
**GABA**	**Polyamines**	0.46 ± 0.30	7.5 ± 2.5	0.00191 ± 0.00061	0.01321 ± 0.00086
**Citrulline**	**Polyamines**	0.88 ± 0.30	43.0 ± 7.7	0.0083 ± 0.0013	0.076 ± 0.052
**Ornithine (Orn)**	**Polyamines**	ND	0.66 ± 0.10	ND	0.0256 ± 0.0024
**Guanine**	**Purine and pyrimidine**	52 ± 20	7.04 ± 0.64	0.158 ± 0.044	0.197 ± 0.098
**Uracil**	**Purine and pyrimidine**	0.121 ± 0.028	0.089 ± 0.057	ND	0.00313 ± 0.00059
**Thymine**	**Purine and pyrimidine**	2.44 ± 0.93	0.38 ± 0.06	0.0062 ± 0.0021	0.0077 ± 0.0046
**Hypoxanthine**	**Purine and pyrimidine**	0.152 ± 0.069	0.121 ± 0.025	ND	ND
**Ribose-5-phosphate**	**Purine and Pyrimidine**	0.73 ± 0.33	1.1 ± 0.4	ND	0.028 ± 0.016
**Shikimic acid**	**Shikimate**	0.0071 ± 0.0034	ND	ND	ND
**Shikimate-3-phosphate**	**Shikimate**	1.24 ± 0.77	0.18 ± 0.12	ND	ND
**Malic acid**	**TCA cycle**	140 ± 45	130 ± 61	0.133 ± 0.078	0.56 ± 0.21
**2-Oxoisovaleric acid**	**TCA cycle**	14.3 ± 7.9	16 ± 12	ND	0.11 ± 0.06
**cis-Aconitic acid**	**TCA cycle**	2.6 ± 1.3	0.99 ± 0.64	ND	ND
**Citric acid**	**TCA cycle**	16.6 ± 8.5	11.0 ± 6.5	ND	ND
**Oxaloacetic acid**	**TCA cycle**	0.035 ± 0.028	ND	ND	ND
**α-ketoglutaric acid**	**TCA cycle**	0.37 ± 0.27	0.066 ± 0.052	ND	0.0136 ± 0.0086
**Isocitric acid**	**TCA cycle**	7.6 ± 4.2	4.7 ± 3.2	ND	ND
**3-Phosphoglyceric acid**	**TCA cycle**	10.4 ± 3.6	2.26 ± 0.78	4.6 ± 2.5	6.5 ± 3.4
**Lactic acid**	**Others**	0.22 ± 0.11	0.21 ± 0.11	0.206 ± 0.081	0.18 ± 0.10

SB: shoot base; EC: embryogenic callus; DC: dry callus; WC: watery callus; ND: not detected; ± indicates the standard deviation where n = 3 biological replicates

**Table 2:** Estimation of cell density (cell/mm^2^) in shoot base and embryogenic and non-embryogenic calli in *B*. *rotunda*.

Tissue	Estimate cell number	Width of observation (μm)	Length of observation (μm)	Area (mm^2^)	Cell density (cell/mm^2^)
**SB**	87 ± 7	104.8 ± 3.8	86 ± 3	0.00905 ± 0.00063	9680 ± 1454
**EC**	152.7 ± 9.5	107.4 ± 1.9	83.0 ± 1.5	0.00891 ± 0.00012	17126 ± 909
**DC**	170 ± 7	107.4 ± 2.2	85.8 ± 3.9	0.00921 ± 0.00027	18312 ± 695
**WC**	44.7 ± 4.9	106.5 ± 2.9	85.91 ± 0.86	0.00915 ± 0.00032	4896 ± 696

SB: shoot base; EC: embryogenic callus; DC: dry callus; WC: watery callus; ± indicates the standard deviation where n = 3 biological replicates

**Fig 1:**
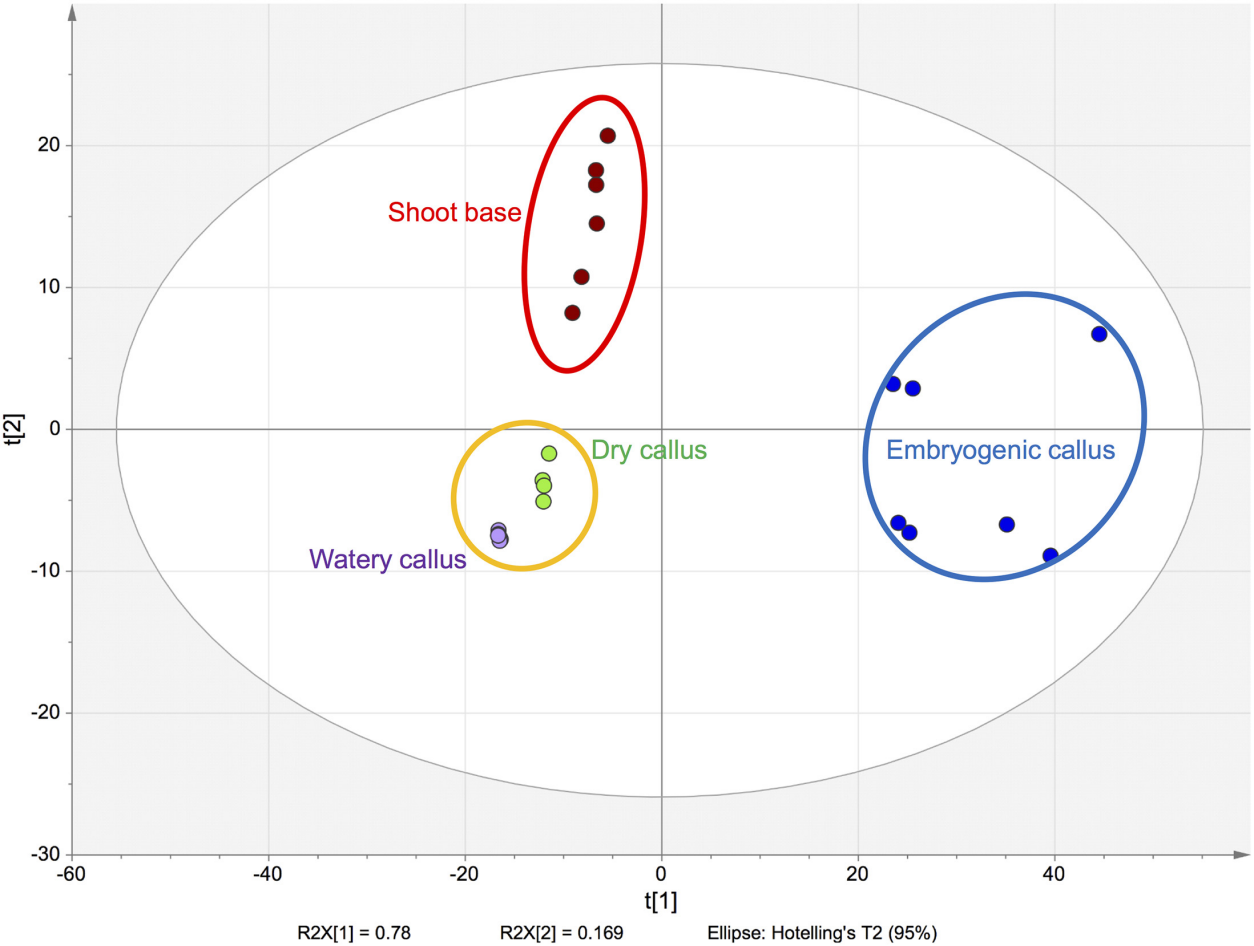
Principal Component Analysis (PCA) plot showing three clusters in callus and explant tissues from *B*. *rotunda* (n = 3 biological replicates). Blue ellipse: embryogenic callus (EC); orange ellipse with green; dry callus (DC) and with purple: watery callus (WC); and red ellipse: shoot base (SB).

**Fig 2:**
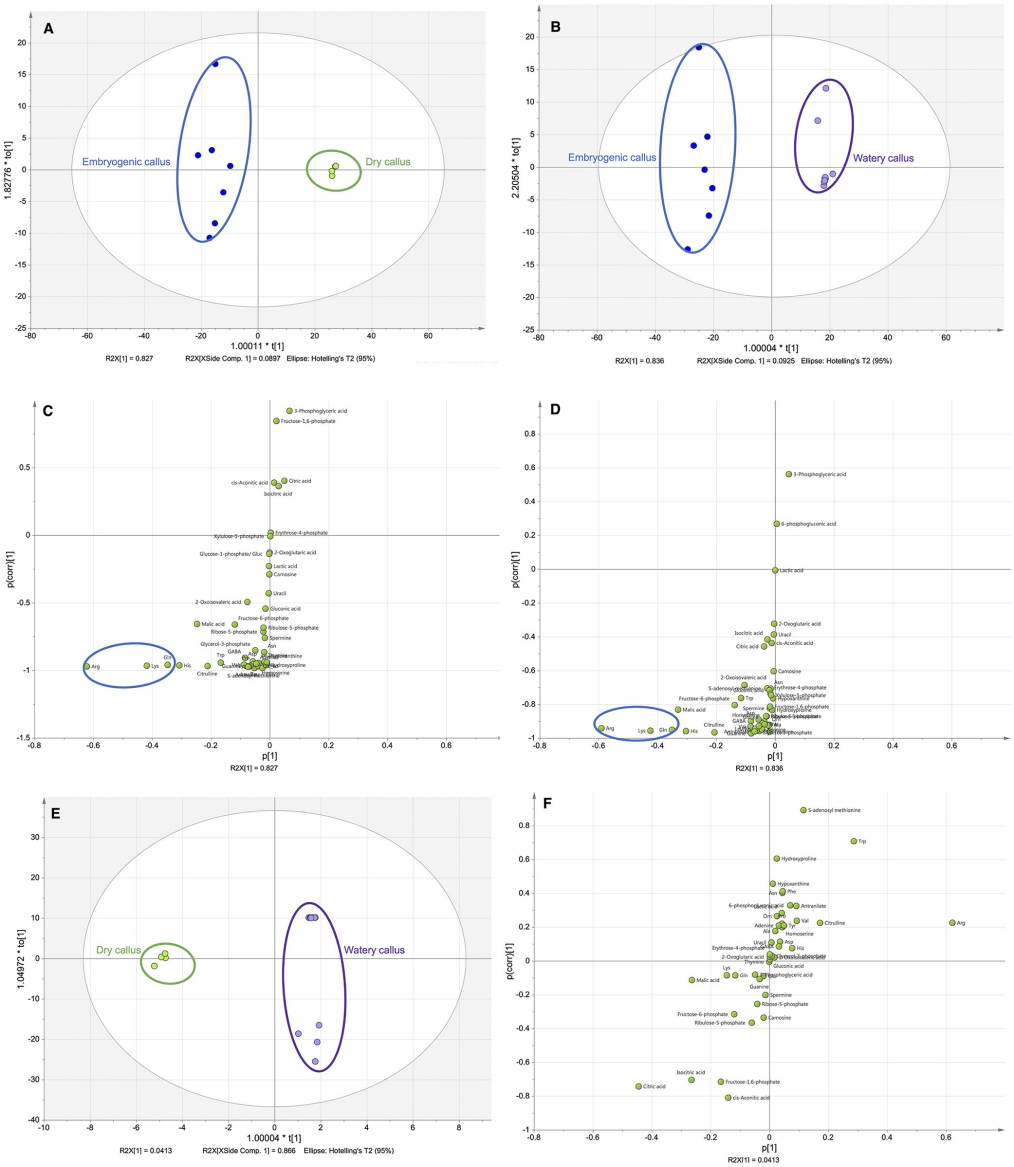
Primary metabolite variables associated with different callus types from *B*. *rotunda* (n = 3 biological replicates). A: Orthogonal Partial Least Square-Discriminant Analysis (OPLS-DA) plot for embryogenic callus (EC) and dry callus (DC); B: OPLS-DA plot for EC and watery callus (WC); C: Blue ellipse in the S plot highlights metabolites associated with EC versus DC with p-value-<0.05, D: Blue ellipse in the S plot highlights metabolites associated with EC versus WC with p-value<0.05, E: OPLS-DA plot for DC and WC; F: S plot showing metabolite comparison between DC and WC.

Multivariate statistical analysis was used to classify the differences between SB, EC, DC and WC of *B*. *rotunda*. Unsupervised principal component analysis (PCA) revealed three major clusters (Fig 1). DC and WC were grouped together, while SB and EC were clustered separately. This suggests that the non-embryogenic callus types, DC and WC, have similar primary metabolite characteristics despite their clearly different morphology (to be discussed below). Detailed relationships of the three callus types (EC, DC and WC) based on primary metabolite profiles is shown in Fig 2. Fig 2A and 2B both show clear distinction between EC and the two non-embryogenic calli, DC and WC, respectively. Subsequently we observed that arginine, glutamine and lysine were most significantly high in EC (Fig 2C and 2D). Both arginine and glutamine have been reported to play major roles in tissue culture proliferation and growth. A study on white pines (*Pinus strobes*) revealed that endogenous levels of glutamine and arginine were associated with early development of zygotic embryos [21]. Another study in Japanese conifer (*Cryptomeria Japonica*) reported high accumulation of glutamine in EC [22]. Glutamine has been reported to be a nitrogen source in carrots [23] and heart vine calli [24] as well as a precursor of other amino acids [25]. Moreover, arginine was reported at higher levels in somatic embryos than in non-embryogenic callus of milk thistle [26]. Arginine is reported as an important precursor for polyamine biosynthesis, via the arginine decarboxylase pathway [27]. It has also been reported that the exogenous application of lysine promotes rice plantlet regeneration [28]. Our result is in concordance with other literature in that high levels of intracellular amino acid lysine found in the EC of *B*. *rotunda* culture may similarly encourage plantlet regeneration via embryogenesis. Specifically comparing DC and WC (Fig 2E and 2F), revealed arginine to be an outlier in the S plot with WC having a relative abundance of about 700 times less than in EC (see Table 1).

**Fig 3:**
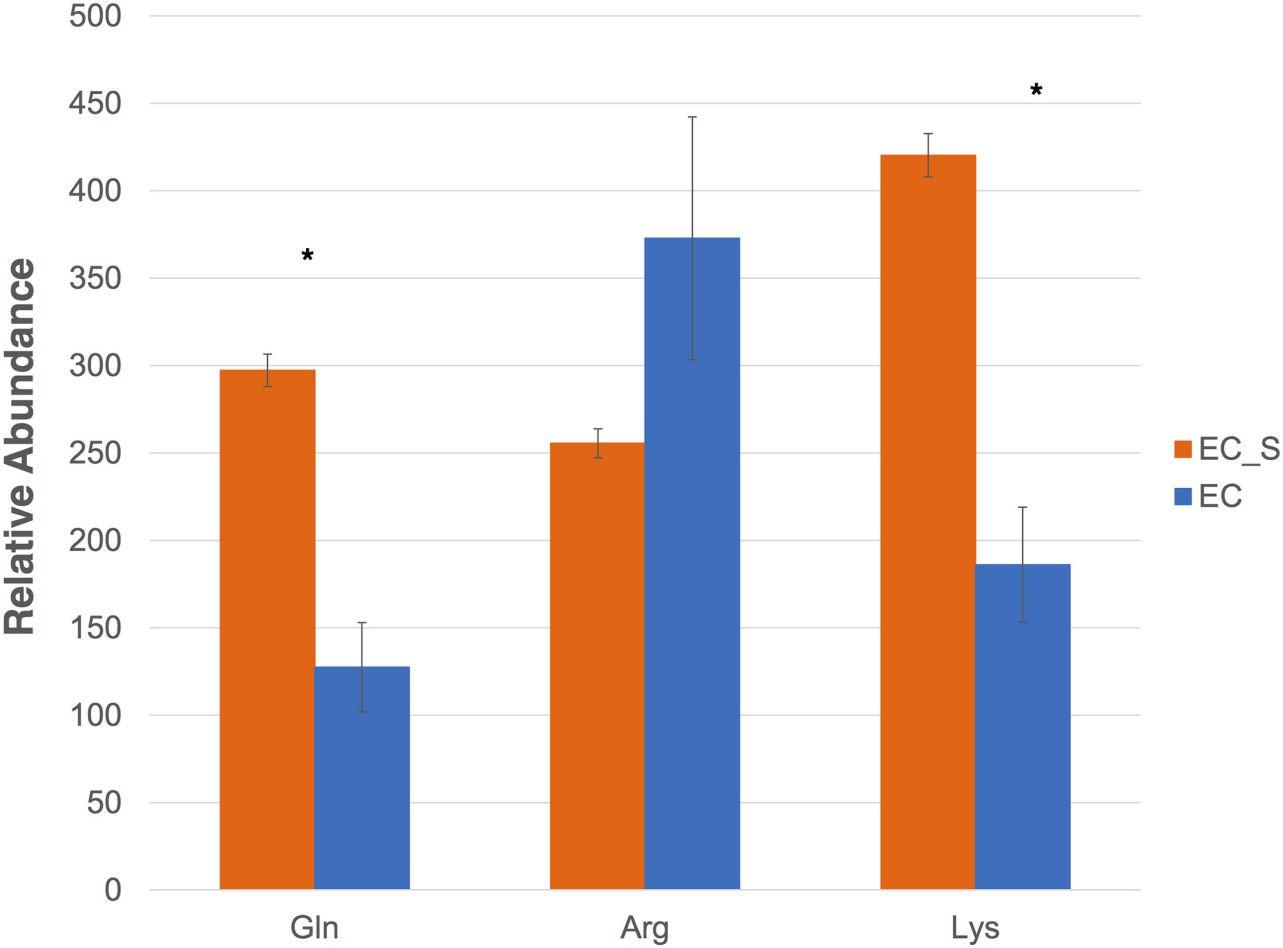
Relative abundance of metabolites markers in embryogenic callus and sieved embryogenic cells (n = 3 biological replicates). EC: Embryogenic callus; EC_S: sieved embryogenic cells. Error bars indicate standard deviation, asterisk represents p-value <0.05 by student T-test.

As embryogenic calli comprised a mixture with a high proportion of embryogenic cells and some non-embryogenic cells, embryogenic calli were sieved in order to enrich samples for embryogenic cells and to confirm the primary metabolite concentrations observed in callus tissue based on morphology. Analysis showed that sieved embryogenic cells (EC_S) had three times higher abundance (p-value < 0.05) of metabolite markers than did embryogenic callus, with the exception of arginine (Fig 3). This confirms the distinct metabolic profiles in embryogenic tissues and suggests that metabolites could be used as indicative markers of embryogenesis in culture cells of *B*. *rotunda*.

### 3.2 Secondary metabolite analysis

**Fig 4:**
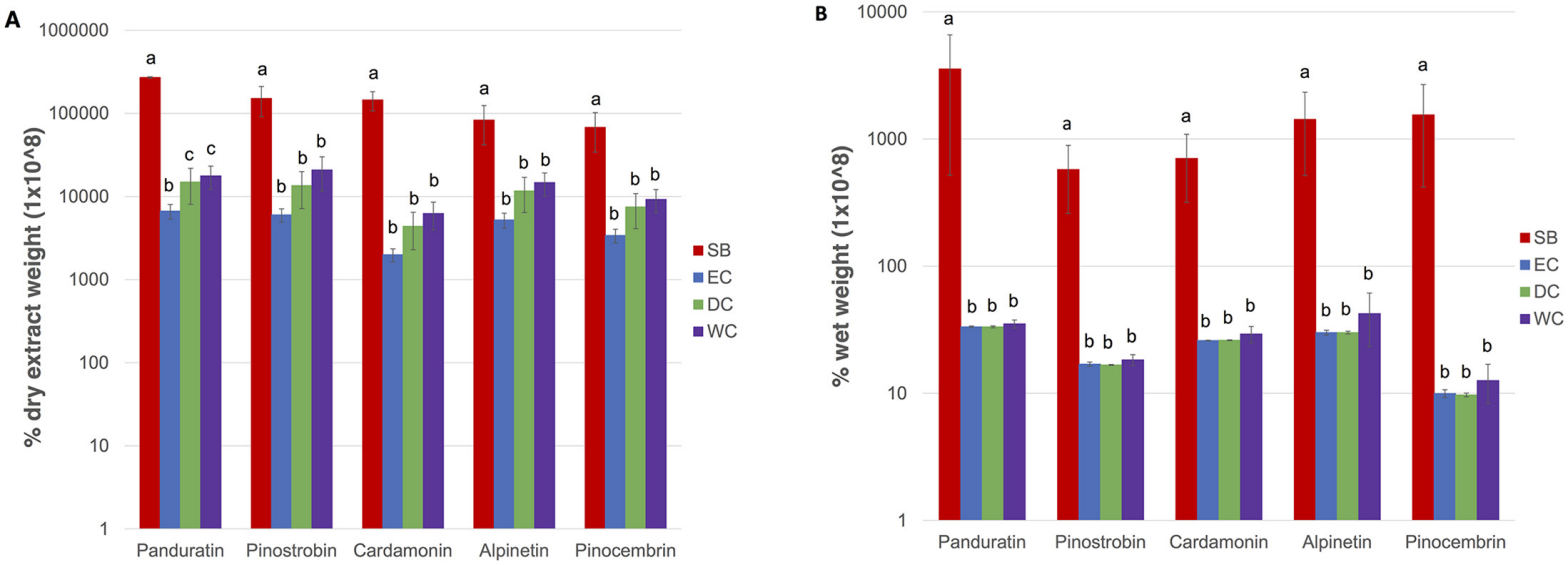
Quantitative analysis of five secondary metabolites in *B*. *rotunda* callus and explant (n = 3 biological replicates). A: values expressed in percent dry weight. B: values expressed in percent wet weight. Red: shoot base (SB); blue: embryogenic callus (EC); green: dry callus (DC); purple: watery callus (WC). Error bars indicate standard deviation and different letters represent significant differences for each metabolites at 95% confidence level by Tukey’s test.

**Fig 5:**
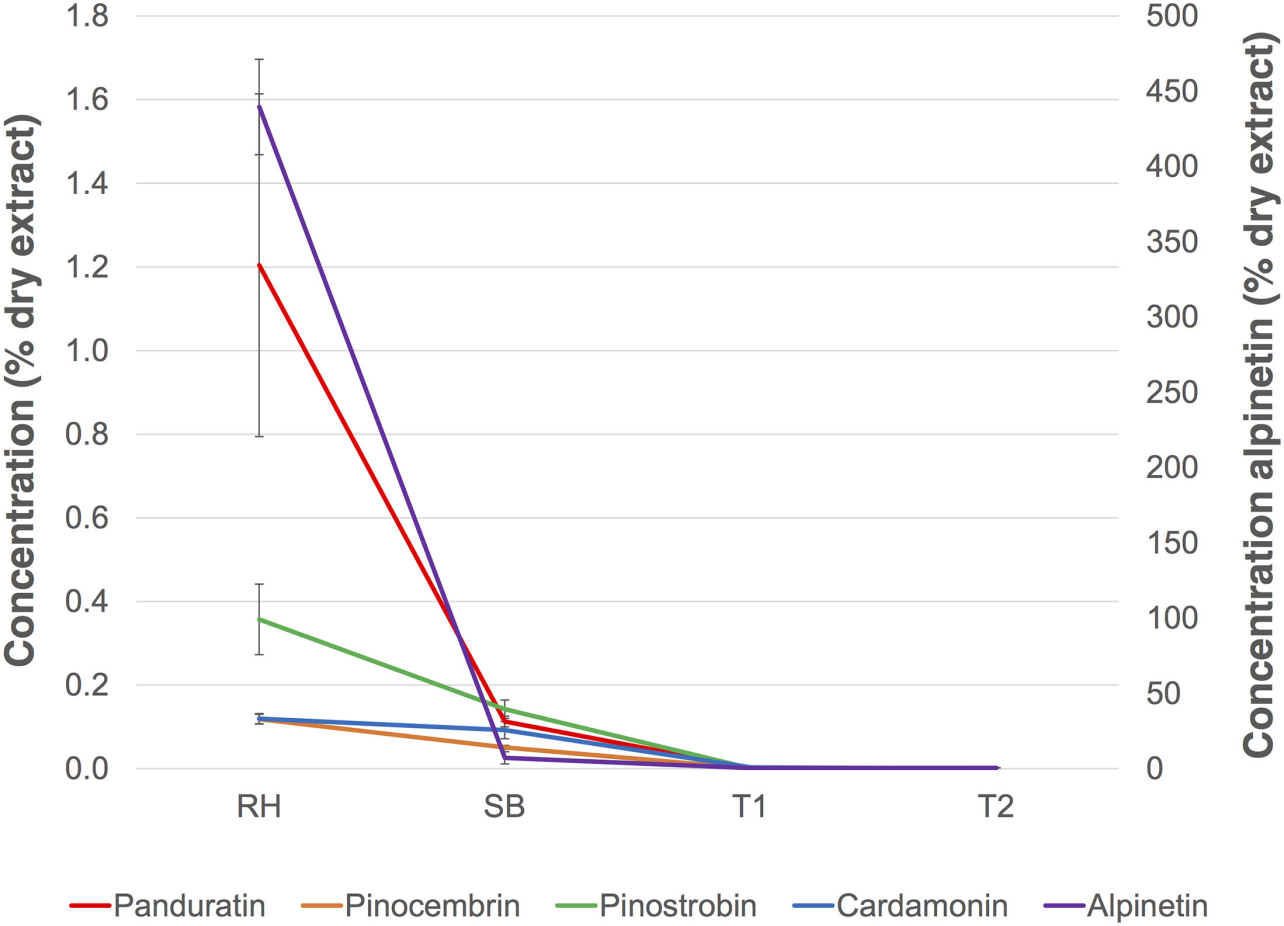
Concentrations (% dry extract) of secondary metabolites in *B*. *rotunda* shoot (n = 3 biological replicates). Error bars indicate standard deviation; RH: rhizome; SB: shoot base; T1: region of shoot 1–5 cm distal from the shoot base; T2: region of shoot 6–10 cm distal from the shoot base.

Five secondary metabolites consisting of three flavonones (pinostrobin, pinocembrin and alpinetin) and two chalcones (panduratin and cardamonin) were quantified according to their various reported medicinal properties. All of the secondary metabolites tested were present at a significant level (p-value <0.05) in more than 10 times greater abundance in shoot base (SB) tissue than in tissues of the three callus type (EC, DC and WC) (Fig 4). This finding is consistent with that of a previous study by Yusuf *et al*. that reported the highest flavonoid concentration in SB tissue followed by mixed callus and finally in cell suspensions of *B*. *rotunda* [29]. The higher secondary metabolite abundance in SB tissue could be due to biosynthesis and storage of compounds in these cells or may arise from diffusion from the rhizomes as the concentration of some flavonones was previously reported [30] to be higher in the rhizome than in the shoot base. Another member of the Zingiberaceae family, *Zingiber officinale Rosc*. has been reported to have a high number of pigmented cells in mature rhizome tissue that stores flavonoids [31]. We investigated this by determining the relative concentrations of the five secondary metabolites under study in rhizomes, in SB and in two shoot sections (T1 and T2) taken from 1–5 cm and 6–10 cm distant from the shoot base (Fig 5). Significantly higher concentrations of all five secondary metabolites were observed in the rhizome, with the concentrations for each decreasing from the shoot base and along the more distal root samples, consistent with the activity of biosynthesis in the rhizome and diffusion of its products along the shoot towards the growing tips.

For *B*. *rotunda*, the very low concentration of secondary metabolites in the calli compared to that in the SB, as similarly observed in suspension cells of *B*. *rotunda* [29] also raises the question of whether the compounds are biosynthesized at relatively low levels in these tissues or are residual from their explant. This has implications for the potential mass propagation through tissue culture of *B*. *rotunda* plants for their secondary metabolites. Transcriptome studies are underway to confirm the apparent lack of activity of the flavonoid biosynthetic pathways in these cultures. Watery callus (WC) had the highest total secondary metabolite concentration of 0.00018% dry extract, while embryogenic callus (EC) had the lowest, at 0.00006% dry extract (Fig 4A). Dry callus (DC) had an intermediate level at 0.00015% of the total dry extract. However, secondary metabolite concentrations based on wet weight were not significantly different across callus types (Fig 4B), suggesting that secondary metabolite production using the fastest growing cell culture type is likely to be the most economical.

The biosynthesis of flavonoids occurs in the phenylpropanoid pathway from primary metabolism of phenylalanine as the precursor. It is interesting to note that EC had low levels of compounds derived from this pathway but the highest abundance of the precursor phenylalanine (Table 1). In contrast, low levels of phenylalanine with higher levels of flavonoids were found in both WC and DC. This suggests that conversion of phenylalanine to flavonoids in EC is minimal, despite activated primary metabolism (Table 1). Thus it seems more likely that the secondary compounds are indeed residual from the SB explant. Similar results have been reported previously for phenolic compounds, in that higher content of phenolic compounds was observed in non-embryogenic callus of chick pea (*Cicer arietinum*) [32], walnut (*Juglans regia*) [33] and alfafa (*Medicago sativa*) [34] than in embryogenic callus.

### 3.3 Auxin

Plant hormone analysis was performed for SB tissue and the three callus types (EC, DC and WC). As most of the classes of hormones (e.g. gibberellins, ABA, cytokinins) were not detected, only auxin data are shown. As has been reported [10, 12], we induced different callus types via the exogenous application of 2,4-D, a well-known plant regulator that initiates growth of calli by regulating the cells to undergo differentiation to form overall calli morphologies. We used the following concentrations for each callus type: dry callus (DC) (4 mg.L^-1^), embryogenic callus (EC) (3 mg.L^-1^), and watery callus (WC) (1 mg.L^-1^). Our results show that the intracellular concentration of 2,4-D was statistically similar in DC (1444 ppb ± 495.3) and WC (1126 ppb ± 136.8). In contrast, 2,4-D was not detected in EC (detection limit of 1 ppb), despite there being an intermediate concentration of 2,4-D in the EC medium. We suggest that a rapid uptake and utilization of this plant growth regulator to induce cell division and promote formation of embryos and shoots could lead to low levels of 2,4-D in EC. When induced with 2,4-D, calli showed totipotency, as reported for *Arabidopsis thaliana* (Raghavan, 2004) and carrots (Komamine et al., 1992). However, the concentration of plant growth regulators included in the culture requires appropriate optimization to avoid adverse effects on callus formation. A study on carrot by Zimmerman *et al*. showed inhibited growth after the globular stage of callusing in the presence of 2,4-D in some carrot cell lines [35].

**Fig 6:**
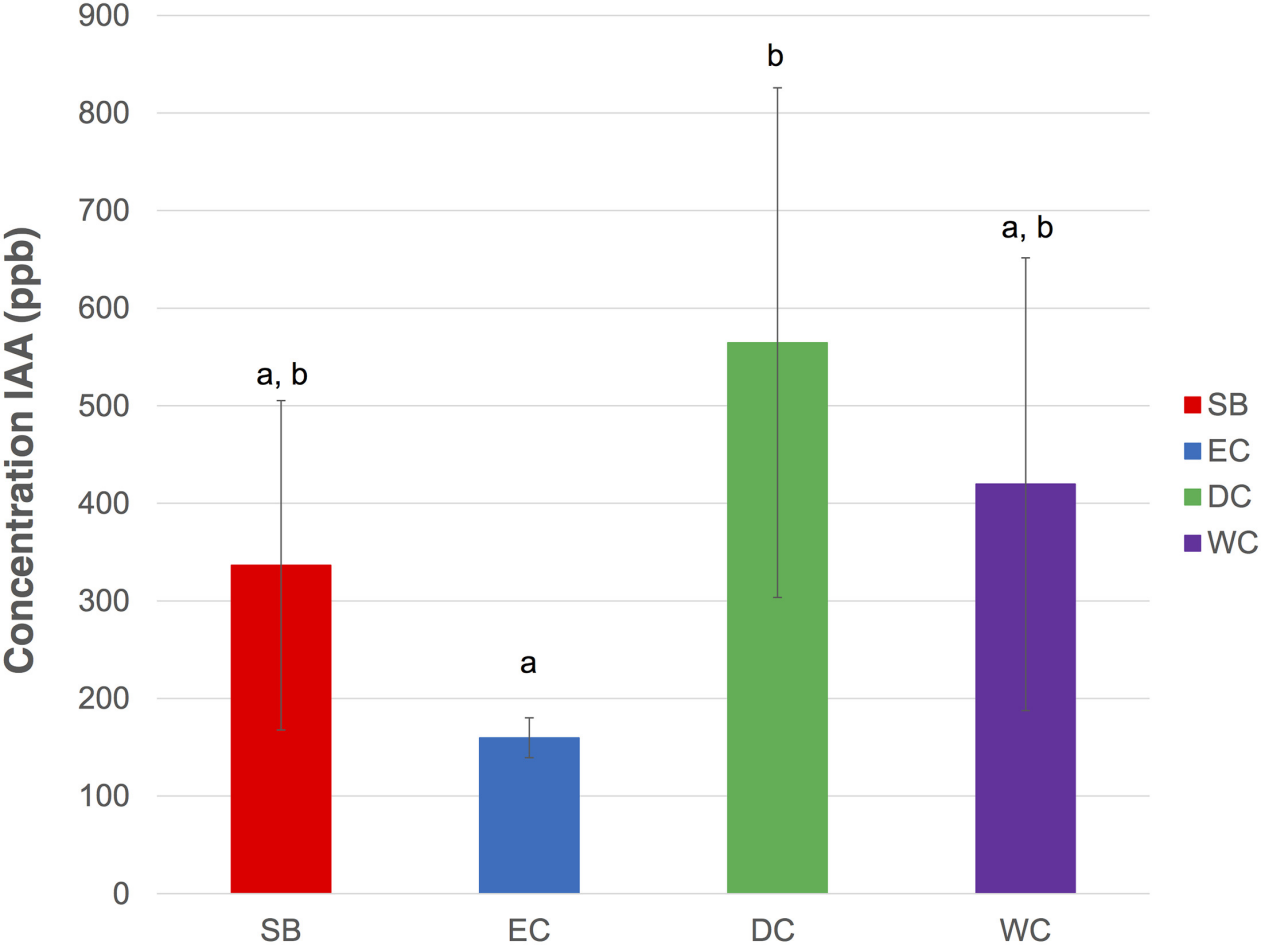
Intracellular hormone IAA concentrations (ppb) in dry extracts of *B*. *rotunda* callus and explant (n = 3 biological replicates). Red: shoot base (SB); blue: embryogenic callus (EC); green: dry callus (DC); purple: watery callus (WC). Error bars indicate standard deviation and different letters represent significant difference at 95% confidence level by Tukey’s test.

The endogenous hormone indole-3-acetic acid (IAA) was observed in decreasing concentrations in the *B*. *rotunda* samples as follows: DC (565 ppb) > WC (420 ppb) > SB (337 ppb) > EC (160 ppb) (Fig 6). The low level of intracellular IAA observed in EC suggests that IAA maybe actively metabolized to stimulate embryogenesis. Several studies have made contradictory reports on levels of IAA in relation to embryogenesis [36–38] which suggests that intracellular IAA levels are not the only deciding factor for embryogenic events. It has been reported that the presence of exogenous 2,4-D may influence the IAA metabolism in carrot cell lines [39, 40]. In the case of *B*. *rotunda*, a moderate level of exogenous 2,4-D leads to formation of embryogenic callus with low intracellular IAA levels.

It is well known that IAA biosynthesis is derived from precursor tryptophan. In *B*. *rotunda*, EC had relatively higher levels of tryptophan than did DC and WC (Table 1). This is in good agreement with the report on the culture of milk thistle, which had a higher level of tryptophan in somatic embryos than did non-embryogenic callus [26]. In rice (*Oryza sativa*) culture, huge quantities of embryogenic calli were obtained in cultured media containing tryptophan [41]. The low levels of tryptophan in *B*. *rotunda* DC and WC suggests that this precursor is almost entirely converted, increasing levels of auxins in the cells, unlike the EC, which has minimal conversion of tryptophan. Moreover, a report on the culture of wild cherry by Sung *et al*.[42] had indicated that high abundance of tryptophan, decreases the endogenous level of IAA in the presence of 2,4-D, a result concurrent with our finding in EC of *B*. *rotunda*.

### 3.4 Histology and morphology

**Fig 7:**
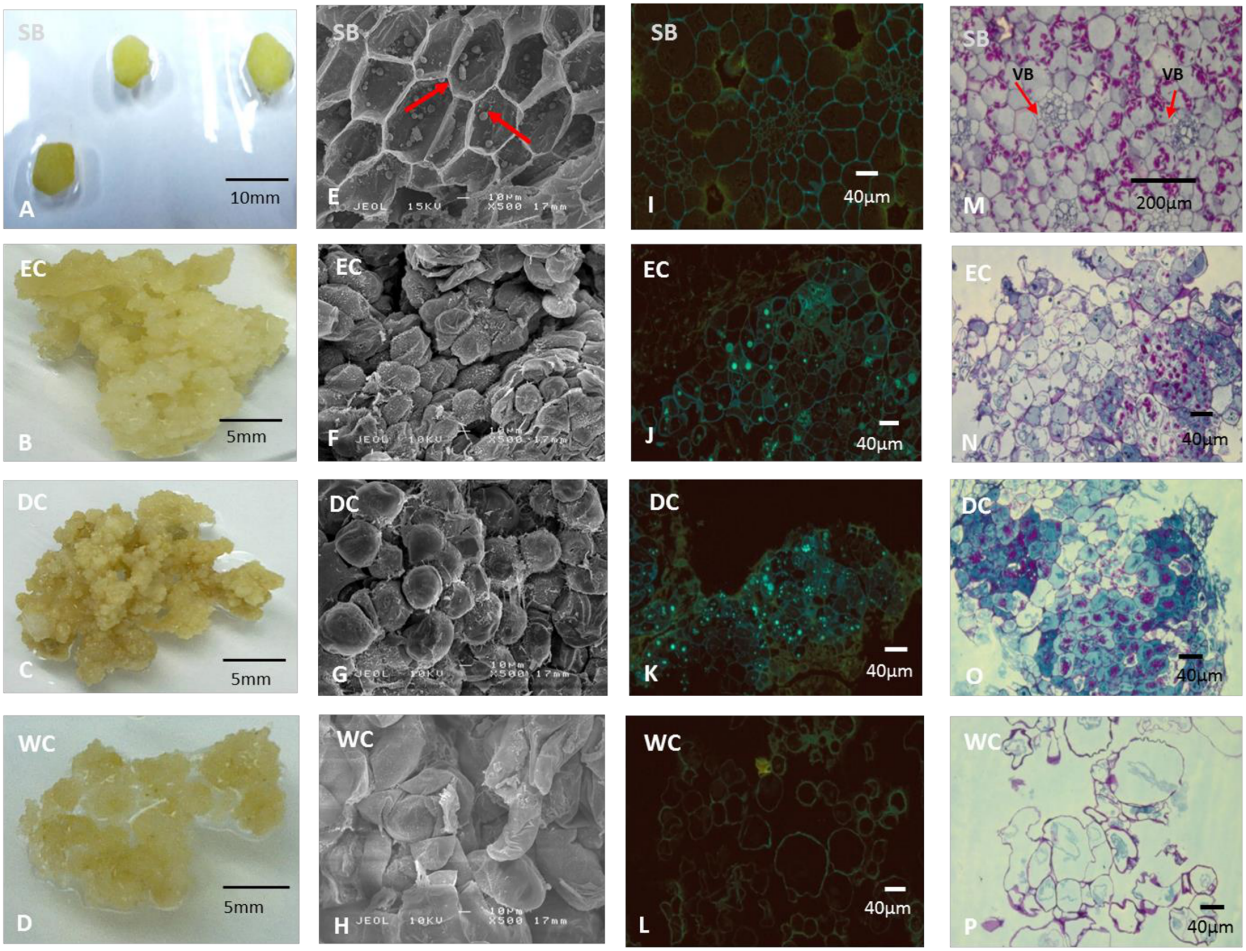
Morphology and histology of *B*. *rotunda* explant and callus. **A-D**: morphology of samples; A: cross section of 1 cm x 1 cm shoot base tissue; B: friable pale yellowish callus; C: compact, dense and dry callus, D: spongy and wet callus; **E-H**: SEM images (100x magnification); E: regular-shaped and -sized cells with arrows showing the presence of starch; F: regular-shaped cells with fibrils; G: rounded, compact cells; H: elongated and irregular-shaped cells; **I-L**: morphology of each sample viewed under fluorescent microscopy with diphenylboric acid 2-aminoethylester (DPBA) stain (100x magnification); I: fluorescent yellowish-green lining of cell membrane, J: fluorescent greenish-blue spots observed with yellow lining of cell membrane; K: fluorescent greenish blue spots observed with yellow lining of cell membrane; L: yellowish lining of cell membrane; **M-P**: morphology of each sample viewed under light microscopy with Periodic acid Schiff (PAS) stain (100x magnification); E: organized and compact cells with presence of vascular bundles (VB) and purplish-red starch granules; F: presence of dark blue clusters indicates active cell division and purplish-red starch granules; G: presence of dark blue clusters indicates active cell division and red-purplish starch granules; H: irregular-shaped and -sized cells without starch granules. SB: shoot base; EC: embryogenic callus; DC: dry callus; WC: watery callus.

In order to document the detailed morphology of the SB and the three callus types, microscopy analysis was carried out. We observed that morphologically the EC were pale-yellowish, globular and friable callus while DC were yellowish, friable, nodular and dry (Fig 7B and 7C). Although WC had a yellowish colour similar to that of EC and DC, its morphology was very much different from the other callus types. WC was spongier than either DC or WC and wet in appearance (Fig 7D). At an early stage of callusing, EC and DC are hard to distinguish, but prominent differences are observed at later stages of callusing, when DC becomes hard callus clumps that resist growth upon sub-culture while cells in EC are observed as globular, translucent spheres which differentiate and develop into somatic embryos for germination. Similar morphologies of *B*. *rotunda* EC have been reported elsewhere [10, 12, 29].

Despite being able to differentiate the physical morphologies of each callus type using light microscopy, visualization by scanning electron microscope (SEM) provided better resolution and information about the cell surface. Using SEM, we observed that both EC and DC had more regular shapes and sizes than did WC, which had irregular shapes and sizes (Fig 7F–7H). Moreover, we observed that EC had more fibrils on the cell surface than did DC cells, which were more rounded and compact. The presence of a membranous layer which turned to fibrils on the cell surface of EC suggests that our EC had potential morphogenic capacity. Similar morphology has also been found in EC of kiwifruit [43]. Furthermore, studies in sugarcane [44] and *Citrus* hybrid callus [45] reported that the absence of fibrils on the cell surface observed using SEM, indicated non-embryogenic calli, a result similar to that found in this study for WC in *B*. *rotunda*. The presence of fibrils, which possibly derived from pectins, has been reported to play a role in cell-to-cell adhesion and in the control of cell wall ionic status and porosity [43]. In addition, Baluska *et al*. (2003) reported the function of pectin oligosacharride fragments released from cell walls as signaling molecules in regulation of overall developmental processes. Fig 7E, showed that the cells of the SB were organized and, regularly-shaped and -sized and that some cells were starting to accumulate starch granules. The presence of starch granules further supports the role of SB as an energy provider through cell metabolism of carbohydrates. Concurrent findings of starch granules have been reported in the rhizomes of mango ginger [46] and the rhizomes of switchgrass [47].

The fluorescent microscopy study was conducted using diphenylboric acid 2-aminoethylester (DPBA) stain to make visible flavonoid abundance and localization in cells. Our results showed the presence of a thin yellowish-green lining of cell membranes across all four samples (Fig 7I–7L). However, only EC and DC showed the presence of fluorescent greenish-blue spots (Fig 7J and 7K) indicating the presence of localized flavonoids, while no fluorescence was observed in WC (Fig 7L). However, this evidence of DPBA staining is in contrast to the evidence of the abundance of the secondary metabolites as measured by UPLC-MS, where watery callus (WC) had the highest concentrations (Fig 4A). This apparent contradiction may be due to different specificity of the DPBA dye for the particular flavonoids in *B*. *rotunda* as DBPA has been reported to have different affinity levels for various flavonoids, for example it has been found to be unable to detect anthocyanin [48–51].

Histological examinations were also performed using light microscopy on the initial explant (shoot base) and the three types of callus of *B*. *rotunda* stained with Periodic acid Schiff (PAS) reagent to view starch reserves. We observed that EC and DC had dense cells with prominent dark blue clusters indicating regions of active cell division (Fig 7N and 7O) despite the fact that only EC leads to further plant development. In EC of oil palm [52] and date palm [53], the meristematic regions were stained dark blue with PAS reagent. In comparison, *B*. *rotunda* WC had cells with irregular shapes and sizes and lacked blue clusters, indicating that these cells were not actively dividing. Moreover, the presence of purplish-red spots in SB, EC and DC indicated the presence of starch grains. In contrast, no starch granules were observed in calli using SEM, perhaps due to the early stage of development of starch granules. Studies of somatic embryos of date palm [53] and embryogenic callus of banana [54] also revealed similarly coloured structures as starch reserves. The SB tissue of *B*. *rotunda* also showed the presence of vascular bundles surrounded with parenchyma cells (Fig 7M).

## 4. Conclusion

We have profiled the primary and secondary metabolites and hormones in shoot base (SB), embryogenic callus (EC) and non-embryogenic (DC and WC) calli of *B*. *rotunda*. EC in *B*. *rotunda* showed high levels of primary metabolites in general, especially glutamine, arginine and lysine. Specific isolation of cells by sieving confirmed the elevated levels of these metabolites in embryogenic cells of *B*. *rotunda*. All calli had significantly lower concentrations of secondary metabolites than did SB, with EC having the lowest abundance. On the other hand, DC had the highest intracellular level of auxins followed by WC, SB and EC. The characterization of the *B*. *rotunda* calli by SEM showed that EC had more fibrils on the cell surface and bright fluorescent spots were observed after DPBA staining. Using PAS staining, SB, EC and DC were observed to have starch reserves, in contrast to WC. The very low level of secondary metabolites present in callus tissues and the accumulation of biosynthetic precursors, especially in EC, suggests that these compounds may be residual from their explant tissue. While WC had the highest concentration of secondary metabolites by UPLC-MS, histological profiling indicated that these cells were non-proliferative, lacking in nuclei and localization of starch and secondary metabolite biosynthesis. Transcriptomic studies of the flavonoid biosynthetic pathway may provide insight into the relatively low levels of secondary metabolites observed in this study, and its implications for possible culture production of these compounds. Future work may also investigate the utility of primary metabolite analysis as an indicator of embryogenic tissue.

## Supporting Information

S1 Table Optimized multiple reaction monitoring (MRM) conditions for primary and secondary metabolite and hormone analysis using UPLC-MS. (DOCX)
