# Supplementary material for: Amino Acid and Secondary Metabolite Production in Embryogenic and Non-Embryogenic Callus of Fingerroot Ginger (Boesenbergia rotunda)
Source: PLoS One. 2016 Jun 3;11(6):e0156714. doi: 10.1371/journal.pone.0156714 (PMC4892522; doi:10.1371/journal.pone.0156714)
Supplement: S1 Table — (DOCX) [file pone.0156714.s001.docx]

**Supplementary Table 1**

Pure authentic standards for all compounds were obtained from Sigma Aldrich and OIChem Ltd and used as is.

| **Primary Metabolites** |  |  |  |  |  |
| --- | --- | --- | --- | --- | --- |
| **Metabolites** | **Mode** | **MRM (m/z) (Parent > Daugther)** | **Dwell (s)** | **Cone (V)** | **Collision Energy (eV)** |
| **Glycine (Gly)** | Positive | 76.3 > 30.0 | 0.004 | 17 | 6 |
| **Homoserine** | Positive | 120.0 > 74.0 | 0.004 | 20 | 12 |
| **Glutamine (Gln)** | Positive | 146.8 > 84.0 | 0.004 | 40 | 16 |
| **Histidine His)** | Positive | 155.9 > 110.0 | 0.004 | 22 | 12 |
| **S-adenosyl methionine** | Positive | 399.4 > 136.0 | 0.004 | 28 | 28 |
| **Spermine** | Positive | 203.4 > 111.9 | 0.004 | 28 | 18 |
| **Arginine (Arg)** | Positive | 174.9 > 70.0 | 0.004 | 20 | 24 |
| **Alanine (Ala)** | Positive | 89.9 > 44.0 | 0.004 | 12 | 14 |
| **Asparagine (Asn)** | Positive | 133.8 > 70.0 | 0.004 | 26 | 14 |
| **Aspartic acid (Asp)** | Positive | 133.8 > 74.0 | 0.004 | 24 | 12 |
| **Glutamic acid (Glu)** | Positive | 148.8 > 65.0 | 0.004 | 46 | 24 |
| **Serine** | Positive | 106.9 > 60.9 | 0.004 | 36 | 16 |
| **Proline (Pro)** | Positive | 116.8 > 70.0 | 0.004 | 20 | 14 |
| **Phenylalanine (Phe)** | Positive | 166.9 > 120.0 | 0.004 | 14 | 10 |
| **Valine (Val)** | Positive | 117.9 > 72.1 | 0.004 | 16 | 14 |
| **Tyrosine (Tyr)** | Positive | 181.9 > 122.9 | 0.004 | 18 | 22 |
| **Trptophan (Trp)** | Positive | 204.9 > 146.0 | 0.004 | 20 | 18 |
| **Hydroxyproline** | Positive | 131.9 > 68.8 | 0.004 | 20 | 8 |
| **Lysine (Lys)** | Positive | 146.9 > 84.0 | 0.004 | 20 | 16 |
| **Methionine (Met)** | Positive | 150.9 > 61.0 | 0.004 | 16 | 20 |
| **Antranilate** | Positive | 137.9 > 92.1 | 0.004 | 14 | 22 |
| **Adenine** | Positive | 135.9 > 91.3 | 0.004 | 36 | 26 |
| **Creatine** | Positive | 131.9 > 90.0 | 0.004 | 22 | 14 |
| **Glycerol-3-phosphate** | Negative | 170.9 > 78.9 | 0.011 | 22 | 12 |
| **Fructose-6-phosphate** | Negative | 258.9 > 96.9 | 0.011 | 22 | 16 |
| **Fructose-1,6-phosphate** | Negative | 338.8 > 96.9 | 0.011 | 26 | 16 |
| **Gluconic acid** | Negative | 194.8 > 128.9 | 0.014 | 20 | 12 |
| **Erythrose-4-phosphate** | Negative | 198.9 > 96.9 | 0.011 | 22 | 12 |
| **Xylulose-5-phosphate** | Negative | 228.9 > 78.8 | 0.011 | 18 | 26 |
| **Ribulose-5-phosphate** | Negative | 228.9 > 96.9 | 0.011 | 20 | 14 |
| **6-phosphogluconic acid** | Negative | 274.9 > 96.9 | 0.011 | 24 | 16 |
| **Putresine** | Positive | 89.4 > 72.1 | 0.004 | 60 | 8 |
| **GABA** | Positive | 103.8 > 87.0 | 0.004 | 20 | 10 |
| **Citrulline** | Positive | 175.9 > 70.0 | 0.004 | 16 | 24 |
| **Ornithine (Orn)** | Positive | 132.9 > 70.0 | 0.004 | 14 | 16 |
| **Guanine** | Positive | 151.9 > 110.9 | 0.004 | 26 | 8 |
| **Uracil** | Positive | 112.9 > 70.0 | 0.004 | 12 | 8 |
| **Thymine** | Positive | 127.9 > 69.0 | 0.004 | 26 | 14 |
| **Hypoxanthine** | Positive | 137.4 > 109.8 | 0.004 | 48 | 18 |
| **Ribose-5-phosphate** | Negative | 228.9 > 96.9 | 0.011 | 20 | 14 |
| **Shikimic acid** | Negative | 172.8 > 92.9 | 0.014 | 20 | 10 |
| **Shikimate-3-phosphate** | Negative | 252.9 > 96.9 | 0.011 | 18 | 20 |
| **Malic acid** | Negative | 133.3 > 114.8 | 0.014 | 24 | 14 |
| **2-Oxoisovaleric acid** | Negative | 114.9 > 71.0 | 0.014 | 18 | 9 |
| **cis-Aconitic acid** | Negative | 172.8 > 84.9 | 0.014 | 20 | 12 |
| **Citric acid** | Negative | 190.8 > 110.9 | 0.014 | 22 | 12 |
| **Oxaloacetic acid** | Negative | 132.9 > 75.0 | 0.014 | 52 | 14 |
| **α-ketoglutaric acid** | Negative | 144.9 > 101.0, 57.0 | 0.014 | 20 | 12, 12 |
| **Isocitric acid** | Negative | 191.9 > 110.9 | 0.014 | 20 | 14 |
| **3-Phosphoglyceric acid** | Negative | 180.8 > 96.9 | 0.011 | 18 | 16 |
| **Lactic acid** | Negative | 89.0 > 43.0 | 0.014 | 20 | 8 |

| **Secondary Metabolites** | |  |  |  |  |
| --- | --- | --- | --- | --- | --- |
| **Metabolites** | **Mode** | **MRM (m/z) (Parent > Daugther)** | **Dwell (s)** | **Cone (V)** | **Collision Energy (eV)** |
| **Panduratin** | Positive | 407.0 > 167.0, 83.0 | 0.163 | 48 | 20, 20 |
| **Pinostrobin** | Positive | 271.0 > 167.0, 103.0 | 0.097 | 46 | 20, 38 |
| **Cardamonin** | Positive | 271.0 > 167.0, 124.0 | 0.063 | 54 | 20, 20 |
| **Alpinetin** | Positive | 271.0 > 167.0, 131.0 | 0.063 | 46 | 20, 20 |
| **Pinocembrin** | Positive | 257.0 > 153.0, 131.0 | 0.063 | 48 | 22, 18 |
|  |  |  |  |  |  |
| **Hormones** |  |  |  |  |  |
| **Metabolites** | **Mode** | **MRM (m/z) (Parent > Daugther)** | **Dwell (s)** | **Cone (V)** | **Collision Energy (eV)** |
| **2,4-D** | Negative | 218.7 > 160.8, 124.8 | 0.020 | 26 | 14, 28 |
| **IAA** | Negative | 173.8 > 158.8, 145.9 | 0.003 | 62 | 18, 14 |
